# Supplementary material for: Focusing attention in working and long-term memory through dissociable mechanisms
Source: Nat Commun. 2025 May 3;16:4126. doi: 10.1038/s41467-025-59359-0 (PMC12049562; doi:10.1038/s41467-025-59359-0)
Supplement: Supplementary file 1 — Reporting Summary [file 41467_2025_59359_MOESM1_ESM.pdf]

nature portfolio

Corresponding author(s): Dongyu Gong, Dr. Dejan Draschkow, Dr. Anna C. Nobre

Last updated by author(s): Mar 29, 2025

Reporting Summary

Nature Portfolio wishes to improve the reproducibility of the work that we publish. This form provides structure for consistency and transparency in reporting. For further information on Nature Portfolio policies, see our Editorial Policies and the Editorial Policy Checklist.

Statistics

For all statistical analyses, confirm that the following items are present in the figure legend, table legend, main text, or Methods section.

n/a	Confirmed	
☐	☒	The exact sample size (*n*) for each experimental group/condition, given as a discrete number and unit of measurement
☒	☐	A statement on whether measurements were taken from distinct samples or whether the same sample was measured repeatedly
☐	☒	The statistical test(s) used AND whether they are one- or two-sided<br>*Only common tests should be described solely by name; describe more complex techniques in the Methods section.*
☒	☐	A description of all covariates tested
☐	☒	A description of any assumptions or corrections, such as tests of normality and adjustment for multiple comparisons
☐	☒	A full description of the statistical parameters including central tendency (e.g. means) or other basic estimates (e.g. regression coefficient) AND variation (e.g. standard deviation) or associated estimates of uncertainty (e.g. confidence intervals)
☐	☒	For null hypothesis testing, the test statistic (e.g. *F*, *t*, *r*) with confidence intervals, effect sizes, degrees of freedom and *P* value noted<br>*Give P values as exact values whenever suitable.*
☒	☐	For Bayesian analysis, information on the choice of priors and Markov chain Monte Carlo settings
☒	☐	For hierarchical and complex designs, identification of the appropriate level for tests and full reporting of outcomes
☐	☒	Estimates of effect sizes (e.g. Cohen's *d*, Pearson's *r*), indicating how they were calculated

Our web collection on statistics for biologists contains articles on many of the points above.

Software and code

Policy information about availability of computer code

Data collection	Psychophysics Toolbox Version 3<br>EyeLink 1000<br>Psychopy 2023.2.2
Data analysis	MATLAB (R2020b); RStudio (Version 2023.03.0+386); VS Code (Version 1.92.2). Analysis code is available at https://github.com/Daniel-Gong/Orienting-in-WM-and-LTM/.

For manuscripts utilizing custom algorithms or software that are central to the research but not yet described in published literature, software must be made available to editors and reviewers. We strongly encourage code deposition in a community repository (e.g. GitHub). See the Nature Portfolio guidelines for submitting code & software for further information.

Data

Policy information about availability of data

All manuscripts must include a data availability statement. This statement should provide the following information, where applicable:

- Accession codes, unique identifiers, or web links for publicly available datasets
- A description of any restrictions on data availability
- For clinical datasets or third party data, please ensure that the statement adheres to our policy

The raw behavioural and eye-tracking data are publicly available through the Open Science Framework at https://doi.org/10.17605/osf.io/n629s/.

Research involving human participants, their data, or biological material

Policy information about studies with human participants or human data. See also policy information about sex, gender (identity/presentation), and sexual orientation and race, ethnicity and racism.

Reporting on sex and gender	Sex was determined based on self-reporting by filling out participant information at the start of the experiment. Experiment 1 included 23 females and 7 males; Experiment 2 included 27 females and 17 males; Experiment 3 included 33 females and 86 males. However, sex was not considered in study design, and findings don't apply to only one sex.
Reporting on race, ethnicity, or other socially relevant groupings	No socially constructed or socially relevant categorization variables were used in this study.
Population characteristics	Experiment 1 involved 30 participants with a mean age of 25.13 years and a standard deviation of 4.15; Experiment 2 involved 44 participants with a mean age of 25.93 years and a standard deviation of 4.51; Experiment 3 involved 119 participants with a mean age of 28.69 years and a standard deviation of 6.04. All participants had normal or corrected-to-normal vision. For Experiments 1 and 2, nearly all participants were undergraduate or graduate students at the University of Oxford; for Experiment 3, all participants were recruited on the Prolific platform (https://www.prolific.co/). Because all experiments used a within-subjects design, none of the above variables constituted a relevant covariate.
Recruitment	For Experiments 1 and 2, participants were recruited through flyers and an online participant database (SONA) at the University of Oxford; for Experiment 3, participants were recruited on Prolific.
Ethics oversight	This study was approved by the Central University Research Ethics Committee of the University of Oxford.

Note that full information on the approval of the study protocol must also be provided in the manuscript.

Field-specific reporting

Please select the one below that is the best fit for your research. If you are not sure, read the appropriate sections before making your selection.

☐ Life sciences ☒ Behavioural & social sciences ☐ Ecological, evolutionary & environmental sciences

For a reference copy of the document with all sections, see nature.com/documents/nr-reporting-summary-flat.pdf

Behavioural & social sciences study design

All studies must disclose on these points even when the disclosure is negative.

Study description	Quantitative experimental study
Research sample	30 volunteers (23 females, 7 males, M = 25.13 years, SD = 4.15) participated in Experiment 1. 44 volunteers (27 females, 17 males, M = 25.93 years, SD = 4.51) participated in Experiment 2. 119 volunteers (33 females, 86 males, M = 28.69 years, SD = 6.04) participated in Experiment 3. All participants had normal or corrected-to-normal vision. For Experiments 1 and 2, nearly all participants were undergraduate or graduate students at the University of Oxford; for Experiment 3, all participants were recruited on the Prolific platform.
Sampling strategy	In each experiment, we used a within-subjects design with random participant sampling. No statistical methods were used to pre-determine the sample size in Experiment 1, but our sample size was chosen to be similar to those reported in previous publications from the lab that had similar designs. The sample size in Experiment 2 was calculated using G*Power to achieve 90% power for the one-sample t-tests performed to test the significance of perceptual benefits following WM and LTM retrocues. The effect sizes for these comparisons in Experiment 1 were 0.877 and 0.797. We assumed a conservative approach and aimed to power for the detection of a medium effect size (0.5) because we expected that the manipulation in Experiment 2 would lead to a smaller effect due to the incidental nature of the spatial attributes in the task. In Experiment 3, given the assumption that LTM would decay on the second day, we aimed to power for detecting an effect size of 0.3 for perceptual benefits, which led G*power to yield a sample size of 119 participants.
Data collection	For Experiments 1 and 2, behavioural task-responses were collected and stored by a computer. Eyetracking data were collected using an Eyelink 1000 Eyetracker, and stored by another computer. Participants were seated in a dimly lit booth and no others were present besides the participant. The experimenter was aware of experimental conditions during data collection. For Experiment 3, participants were required to use a desktop PC or a laptop to take the study. Participants were asked to sit in a quiet place and make sure they would be undisturbed while taking part in the study.
Timing	April 2022 to May 2024
Data exclusions	Experiment 1: for the testing session, data from the memory recall and perceptual discrimination tasks were analysed separately. During pre-processing, we excluded trials on which RTs were 3 SD above the individual mean across all conditions in either task. After this exclusion step, an average of 98.35% (SD = 0.44%) trials were retained in the analyses.<br>Experiment 2: after excluding memory recall and perceptual discrimination trials on which RTs were 3 SD above the individual mean across all conditions, an average of 98.66% (SD = 0.47%) trials were retained in the analyses. Besides, for eye-tracking analysis, we

only included trials on which horizontal and vertical gaze positions remained within ± 50% from fixation (with 100% denoting the centres of the original item locations at a ± 5° visual angle) throughout the course of the trial, as previous work showed that the gaze bias phenomenon is constituted by a bias in gaze around fixation. For the horizontal channel, data for all 44 participants were used, with an average of 9.6% ± 1.7% (M ± SEM) trials excluded per participant. For the vertical channel, six participants had to be removed due to a high number of excluded trials (> 50%). For the 38 participants retained, an average of 14.6% ± 2.1% trials were excluded. Experiment 3: during pre-processing, we excluded memory retrieval and perceptual discrimination trials with RTs exceeding 3 SD above the individual mean across all conditions. After this procedure, an average of 98.65% (SD = 0.61%) trials were retained in the analyses.

Non-participation	No participants dropped out/declined participation.
Randomization	The study did not contain experimental groups as each task involved a within-subjects design.

Reporting for specific materials, systems and methods

We require information from authors about some types of materials, experimental systems and methods used in many studies. Here, indicate whether each material, system or method listed is relevant to your study. If you are not sure if a list item applies to your research, read the appropriate section before selecting a response.

Materials & experimental systems

n/a	Involved in the study	
☒	☐	Antibodies
☒	☐	Eukaryotic cell lines
☒	☐	Palaeontology and archaeology
☒	☐	Animals and other organisms
☒	☐	Clinical data
☒	☐	Dual use research of concern
☒	☐	Plants

Methods

n/a	Involved in the study	
☒	☐	ChIP-seq
☒	☐	Flow cytometry
☒	☐	MRI-based neuroimaging
